# Sensory neurons that respond to sex and aggregation pheromones in the nymphal cockroach

**DOI:** 10.1038/s41598-020-58816-8

**Published:** 2020-02-06

**Authors:** Kosuke Tateishi, Yukihiro Nishimura, Masayuki Sakuma, Fumio Yokohari, Hidehiro Watanabe

**Affiliations:** 1 Division of Biology, Department of Earth System Science, Fukuoka University, 8-19-1 Nanakuma, Jonan-ku, Fukuoka 814-0180 Japan; 2 Division of Applied Biosciences, Graduate School of Agriculture, Kyoto University, Kitashirakawaoiwake-cho, Sakyo-ku, Kyoto 606-8502 Japan

**Keywords:** Olfactory system, Peripheral nervous system, Sensory processing, Sexual behaviour, Animal physiology

## Abstract

In the common pest cockroach, *Periplaneta americana*, behavioural responses to the sex and aggregation pheromones change in an age-dependent manner. Nymphs are attracted by the aggregation pheromone periplanolide-E (PLD-E) but not by the sex pheromone periplanone-B (PB) in faeces. Adults display prominent behaviours to PB but not to PLD-E. Despite the significant behavioural differences depending on postembryonic developmental stages, peripheral codings of the sex and aggregation pheromones have not been studied in the nymph of any insects as far as we know. In this study, we morphologically and electrophysiologically identified antennal sensilla that respond to PB and PLD-E in nymphal cockroaches. Although nymphs lacked the sex pheromone-responsive *single-walled* B (*sw-*B) sensilla identified in adult males, we found PB-responsive sensory neurons (PB-SNs) within newly identified *sw-*A2 sensilla, which exhibit different shapes but have the same olfactory pores as *sw-*B sensilla. Interestingly, PLD-E-responsive sensory neurons (PLD-E-SNs) were also identified in the same sensillar type, but PB and PLD-E were independently detected by different SNs. Both PB-SNs and PLD-E-SNs showed high sensitivity to their respective pheromones. The hemimetabolous insect nymph has an ability to detect these pheromones, suggesting that behaviours elicited by pheromones might be established in brain centres depending on postembryonic development.

## Introduction

Pheromones are chemical agents that effectively trigger species-specific behaviours to conspecifics, such as aggregation, sexual and social behaviors^1^. As cockroaches are one of the major pests in urban environments, effective control of their behaviour using pheromones has been extensively investigated with regarding to ecological, pharmacological and physiological aspects. Thus, pheromones have been identified in several species of cockroaches^2–5^. In the American cockroach, *Periplaneta americana*, two sex pheromones have been isolated, the subcomponent periplanone-A (PA) and the main component periplanone-B (PB)^6–8^. Adult male cockroaches are strongly attracted by the sex pheromones emitted by adult females, which elicit sexual behaviours^9^, but nymphal cockroaches do not exhibit any behavioural responses to the sex pheromones^10,11^. In the cockroach, adults and nymphs at various developmental stages rest in a group in nature environment^12^. Especially, nymphs are strongly attracted by their faeces^13^. Recently, six attractive compounds, termed periplanolide-A to -F (PLD-A to -F), were identified from dried faeces of *P. americana* and were successfully synthesised^14^. Behavioural experiments showed that each PLD compound strongly and weakly attracts early instar nymphs and adults, respectively^14^. Thus, behaviours in response to the sex and aggregation pheromones are significantly different depending on the postembryonic developmental stages of the cockroach. However, it is still unknown how nymphal cockroaches detect and process these pheromones.

**Figure 1:**
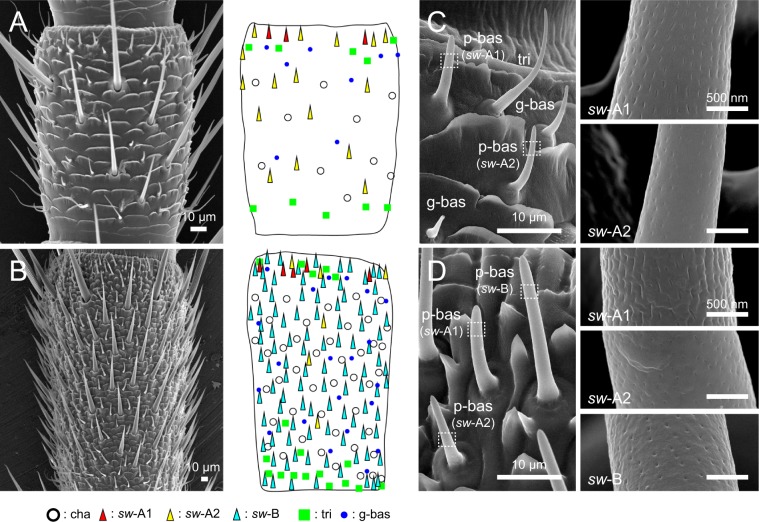
Classification of antennal olfactory sensilla in the nymphal and adult male cockroaches. (**A**) Sensillar distribution on a flagellomere in the distal part of a fourth instar male antenna. A SEM image of the 43rd flagellomere (left) and its schematic drawing (right) denote the sensillar distribution. (**B**) Sensillar distribution on a flagellomere in the distal part of an adult male antenna. A SEM image of the 94th flagellomere (left) and its schematic drawing (right) denote the sensillar distribution. Adult male antenna equips a large number of sex pheromone-responsive *sw*-B sensilla (blue triangles). cha: chaetic sensillum. (**C**) Nymphal olfactory sensilla. The antenna contains perforated basiconic (p-bas), grooved basiconic (g-bas), and trichoid (tri) sensilla. Based on the shapes of olfactory pores, p-bas sensilla further divided into *sw*-A1 and *sw*-A2 sensilla in nymphs (insets in C). (**D**) New classification of adult olfactory sensilla. Based on differences of sensillar lengths and olfactory pore shapes, p-bas sensilla further divided into *sw*-A1, *sw*-A2 and *sw*-B sensilla in adult males (insets in D). In both postembryonic developmental stages, *sw-*A1 sensilla equip with slit-like olfactory pores, whereas *sw*-A2 and *sw*-B sensilla equip with circular pores.

In *P. americana*, neural processing of sex pheromones has been well studied on levels ranging from peripheral to higher brain centres in adult males. Odourants including pheromones are generally detected by olfactory sensory neurons (OSNs) in antennal olfactory sensilla. In the adult, antennal olfactory sensilla are classified into three morphological types: perforated basiconic, grooved basiconic and trichoid sensilla^15^. Among them, perforated basiconic sensilla are further classified on the basis of shaft length into *single walled-*A (*sw-*A) sensilla (Fig. 1; 8–12 µm in length) and *sw-*B sensilla (Fig. 1; 18–28 µm in length)^15–18^. *Sw-*A and *sw-*B sensilla contain two and four OSNs, respectively^15–18^. Both of the OSNs in *sw-*A sensilla detect general odours, but single *sw-*B sensilla contain PA- and PB-responsive sensory neurons (PA-SNs and PB-SNs) in addition to two OSNs that detect general odours^19–22^. PA-SNs and PB-SNs extend their axons into A- and B-glomeruli in the antennal lobe (AL), respectively. Because of the greater number of *sw-*B sensilla on the male antenna, the A- and B-glomeruli are enlarged in males compared with the homologous glomeruli in females^23^. In the A- and B-glomeruli, PA- and PB-SNs synapse onto PA- and PB-responsive projection neurons (PA-PNs and PB-PNs)^24–26^.

Although the antennae of nymphal cockroaches lack *sw-*B sensilla^17,27,28^, previous studies suggested that nymphal cockroaches sense sex pheromones as follows. First, the A- and B-glomeruli are formed during early development, and the volumes of these glomeruli gradually increase at every moult concomitantly with the elongation of the antennae^28–30^. Second, the nymphal antennae exhibit weak but reliable electroantennogram responses to sex pheromones^31–33^. Third, later instar nymphs have PB-PNs that have dendrites in the nymphal B-glomerulus^34^. Finally, a large majority of nymphal *sw-*A sensilla are transformed into *sw-*B sensilla at the final moult, and some OSNs in the nymphal *sw-*A sensilla extend their axon terminals into the A- and B-glomeruli^28^. Therefore, nymphal *sw-*A sensilla may be able to detect sex pheromones. However, the physiological properties of nymphal OSNs are still unclear. Furthermore, neural processing of aggregation pheromones has not been clarified in either nymphal or adult cockroaches.

In this study, morphological and electrophysiological analyses of nymphal antennal sensory systems revealed that male and female nymphal cockroaches can sense both sex and aggregation pheromones via OSNs in specific sensilla with high sensitivities. It is unclear the function of sex pheromone detection ability of nymphal stages because of lack of behavioural bioassay. However, comparisons of pheromone processing between nymphs and adults will provide valuable insights for understanding the neural mechanisms of behaviours elicited by pheromones in insects.

## Results

### Olfactory sensilla of nymphal cockroaches

To characterise the morphological features of antennal olfactory sensilla in nymphal cockroaches, we observed flagella of the distal parts of antennae in fourth instar males (N = 5) and females (N = 5) with a field emission scanning electron microscope (SEM) and compared with those of the adult male antennae (N = 5) (Fig. 1). In the cockroach, the antenna is composed of the scape, pedicel, and flagellum, and the flagellum of the fourth instars and adult males consist of approximately 70 and 140 flagellomeres, respectively^27^. On the flagellum of nymphal antennae, antennal sensilla were sparsely distributed compared with those of adult males (Fig. 1A,B)^17,28^. We distinctively observed perforated basiconic, grooved basiconic, and trichoid sensilla on nymphal antennae (Fig. 1A,C), which have been identified as olfactory sensilla in adult antennae (Fig. 1B,D)^15,17^. Based on the differences of sensillar lengths, adult males have two types of perforated basiconic sensilla, *sw-*A (8–12 µm in length) and *sw-*B sensilla (18–28 µm in length) (Fig. 1D)^17^. Although nymphal antennae lacked the *sw-*B sensilla, as reported previously (Fig. 1A)^17,27,28^, we found that nymphal *sw-*A sensilla could be classified into *sw-*A1 and *sw-*A2 sensilla on the basis of olfactory pore shapes (Fig. 1C). The *sw-*A1 sensilla had larger basal diameters (~3 µm) and were equipped with slit-like olfactory pores on their cuticular apparatus, whereas the *sw-*A2 sensilla had smaller basal diameters (~2 µm) and were equipped with circular olfactory pores (Fig. 1C). A few *sw-*A1 sensilla were present in the distal region of each nymphal flagellomere, whereas many *sw-*A2 sensilla were distributed throughout the flagellomere (Fig. 1A). No sex differences were observed in the sensillar distribution pattern in nymphs. We also observed shapes of olfactory pores of adult *sw-*A and *sw-*B sensilla. We found that the morphological criteria of *sw-*A1 and *sw-*A2 sensilla in nymphs were also applicable in adult *sw-*A sensilla (Fig. 1D). Interestingly, all observed *sw-*B sensilla equipped with circular pores as same as the *sw-*A2 sensilla (Fig. 1D). In adults, *sw*-A1 sensilla selectively distributed in the distal region of each flagellomere, suggesting loci of *sw*-A1 sensilla were maintained during postembryonic development (Fig. 1A,B). In adult males, both *sw-*A2 and *sw*-B sensilla were sparsely and densely distributed throughout a given flagellomere, respectively (Fig. 1B).

### Responses of nymphal sensilla to sex and aggregation pheromones

**Figure 2:**
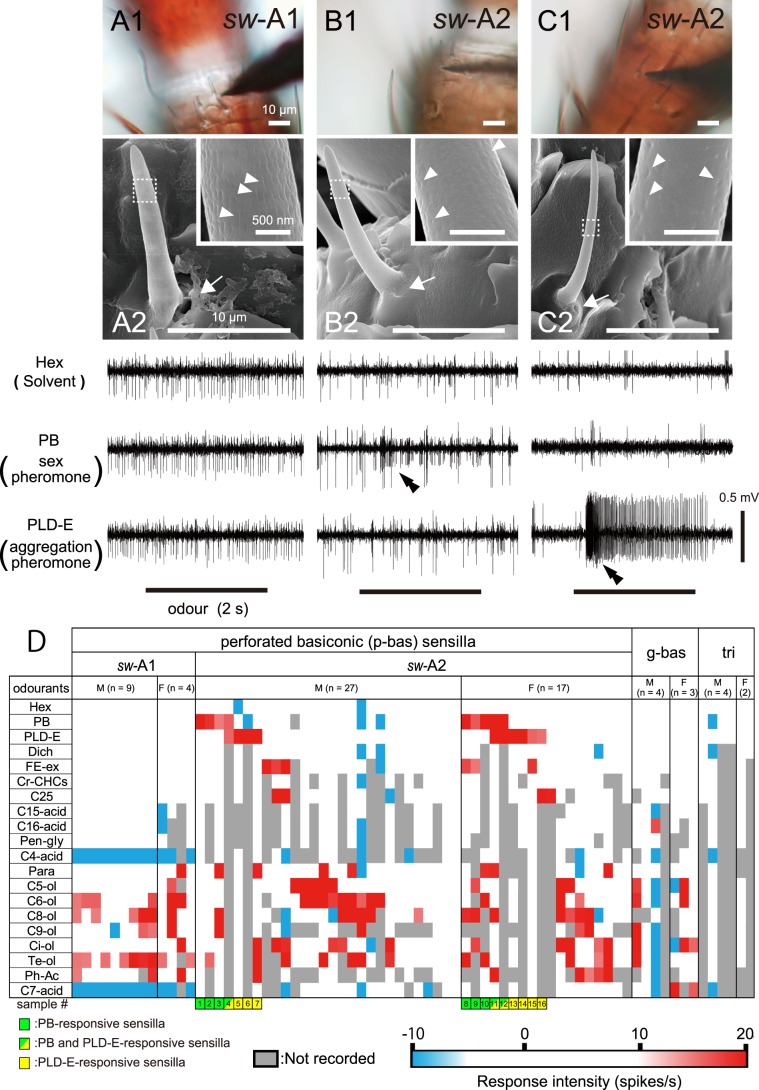
Olfactory responses of single sensilla on the nymphal antennae. (**A**–**C**) Typical responses and morphological features of *sw-*A1 (**A**), PB-responsive *sw-*A2 (**B**), and PLD-E-responsive *sw-*A2 sensilla (**C**). Because we could not identify sensillar types of *sw*-A sensilla under the light microscope (A1–C1), recorded sensilla were observed using SEM in detail (A2–C2). SEM observation reveals olfactory pores of recorded sensilla (arrowheads in insets of A2–C2). Arrows in A2–C2 indicate the insertion sites of electrodes. Sex and aggregation pheromones elicited small-amplitude and large-amplitude spikes in single *sw-*A2 sensilla, respectively (double arrowheads in B,C). (**D**) Response intensities of single sensilla to tested odourants. Response intensity to a given odourant is colour-coded according to the “R − R0” value (see Material and Methods). Excitatory and inhibitory responses are shown as warm and cold colours, respectively. 16 *sw-*A2 sensilla which exhibited excitatory responses to PB and/or PLD-E are numbered (sample numbers below the heatmap). M: male, F: female. g-bas: grooved basiconic sensilla. tri: trichoid sensilla.

To identify the olfactory sensilla that detect sex and aggregation pheromones, we recorded responses from a total of 213 olfactory sensilla of fourth instar nymphs of both sexes using the single sensillum recording (SSR) method. We successfully recorded olfactory responses from 57 perforated basiconic, seven grooved basiconic, and six trichoid sensilla on male and female nymphal antennae. In each SSR, several different shapes of spikes that were discharged from different OSNs in a single sensillum were concurrently recorded (Fig. 2A–C). Based on these spike shapes, we identified two, three, and two OSNs in single perforated basiconic, grooved basiconic, and trichoid sensilla, respectively. To classify recorded perforated basiconic sensilla into *sw-*A1 and *sw-*A2 sensilla, recorded sensilla were further observed in detail with SEM (Fig. 2A2–C2). In the result, we successfully recorded olfactory responses of 13 *sw*-A1 and 44 *sw*-A2 sensilla in this study. In both *sw*-A1 and *sw*-A2 sensilla, large- and small-amplitude spikes were spontaneously discharged (Fig. 2A–C). The averaged spontaneous firing rate of 13 *sw*-A1 sensilla (13.1 Hz) was higher than that of 44 *sw*-A2 sensilla (9.6 Hz). As shown in Fig. 2B, PB activated the small-amplitude spike OSN but not the large-amplitude spike OSN in a *sw-*A2 sensillum. Thus, each *sw-*A sensillum was innervated by paired OSNs with different response profiles. Although the spike amplitudes were distinguishable in several traces, the spikes were not easily distinguishable in many recorded sensilla. Therefore, we first characterised recorded sensilla in terms of the total number of spikes per sensillum and then calculated the increase in spike frequency from the spontaneous level (Fig. 2D).

In SSRs, we initially attempted to record the responses to 2 ng of PB, 2 ng of PLD-E and faecal extract, which contains the equivalent to 2 ng of PLD-E (Table 1)^14^. These pheromones elicited a strong excitatory response from some *sw-*A2 sensilla but did not elicit any response from other morphological types of sensilla (Fig. 2B–D). Thereafter, we increased the number of test odourants included six reported attractants of nymphal cockroaches^5,35–37^ and eight chemically diverse odourants that are frequently used for classification of OSN types (Table 1)^20,22,38^. Each of the general odours largely activated the *sw-*A1, *sw-*A2 and grooved basiconic sensilla (Fig. 2D). Most of the *sw-*A1 sensilla exhibited strong inhibitory responses to C4- and C7-acids, which are known to elicit excitatory responses from OSNs in the grooved basiconic sensilla of adults^20,22^. Among the six attractive odourants, pentacosane (C25) selectively activated a small subset of *sw-*A2 sensilla. Because pheromones and attractive odours were selectively detected by *sw-*A2 sensilla, we focused on the physiological properties of this type of sensillum in subsequent studies.

**Table 1:** Pheromones and odourants used in this study.

Odourants	Abbreviations	Purity (%)	Solvent	Concentration	Ref.
**Solvent**					
n-hexane	Hex	≥99%			
dichloromethane	Dich	≥99%			
n-paraffin-oil	Para				
**Sex pheromone**					
periplanone-B	PB		Hex	2 ng/µl	^50^
**Aggregation pheromone**					
periplanolide-E	PLD-E		Hex	2 ng/µl	^14^
faecal extract	FE-ex		Dich	16.6 µl	^13^
**Aggregation-inducing substances**					
crude extract of CHCs	Cr-CHCs		Dich	10 µl	^35,36^
pentacosane	C25	≥97%	Dich	20 µg/µl	^35,36^
pentadecanoic acid	C15-acid	≥99%	Dich	10 µg/µl	^5^
hexadecanoic acid	C16-acid	≥99%	Dich	10 µg/µl	^5^
pentaethylene glycol	Pen-gly	≥90%	Dich	10 µg/µl	^5^
butyric acid	C4-acid	≥98%	Para	10 mM	^37^
**General odours**					
n-pentanol	C5-ol	≥98%	Para	10 mM	^20,22^
n-hexanol	C6-ol	≥97%	Para	10 mM	^20,22^
n-octanol	C8-ol	≥98%	Para	10 mM	^20,22^
n-nonanol	C9-ol	≥99%	Para	10 mM	^20,22^
cineol	Ci-ol	≥85%	Para	10 mM	^20,22^
α-terpineol	Te-ol	≥80%	Para	10 mM	^20,22^
phenyl acetate	Ph-Ac	≥98%	Para	10 mM	^20,22^
heptanoic acid	C7-acid	≥98%	Para	10 mM	^20,22^

Among 44 recorded *sw-*A2 sensilla, 16 *sw-*A2 sensilla exhibited phasic-tonic responses to PB and/or PLD-E (numbered sensilla in Fig. 2D), and another 28 sensilla did not respond to these pheromones (Fig. 2D). That is, nymphal antennae contained both pheromone-responsive *sw-*A2 and pheromone-unresponsive *sw-*A2 sensilla. The pheromone-responsive *sw*-A2 sensilla were narrowly tuned to the pheromones and a few general odours, whereas the pheromone-unresponsive *sw-*A2 sensilla broadly responded to many general odours. The response spectra of pheromone-unresponsive *sw-*A2 sensilla differed from those of *sw-*A1 sensilla. For example, C4- and C7-acids generally elicited inhibitory responses in *sw-*A1 sensilla, but these acids did not elicit any responses in *sw-*A2 sensilla. These results indicate that OSNs in *sw-*A1 and *sw-*A2 sensilla express distinct repertories of olfactory receptors. Both PB- and PLD-E-responsive *sw-*A2 sensilla were commonly present in both male and female nymphs (Fig. 2D). Therefore, nymphal cockroaches can sense sex pheromones irrespective of their sexes.

**Figure 3:**
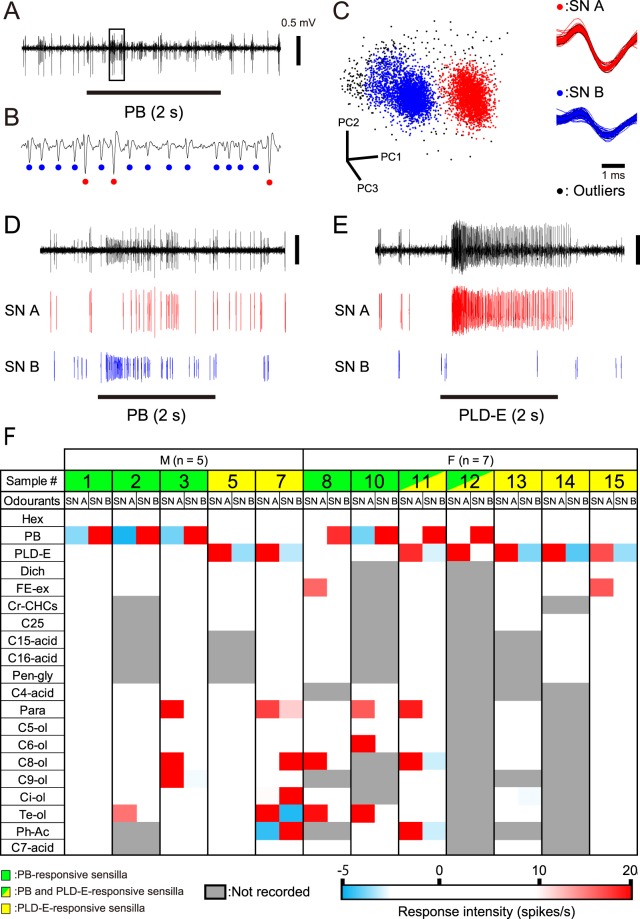
Olfactory responses of OSNs in single pheromone-responsive *sw-*A2 sensilla. (**A**) The response of a *sw-*A2 sensillum to PB. (**B**) Expanded electrophysiological trace shown in A. The trace shows small-amplitude spikes (blue dots) and large-amplitude spikes (red dots). (**C**) Identification of two OSNs in single *sw-*A2 sensilla. Each of spikes is plotted based on the first three principal components (PC1-PC3) obtained from the principal component analysis using the spike amplitudes and durations (left). Cluster analysis identified two groups of spikes (red and blue dots). Outliers of the cluster analysis are shown as black dots in the 3D-space and removed from the subsequent analyses. The spike waveforms of red and blue dots are superimposed (right), and large-amplitude spikes of SN A and small-amplitude spikes of SN B are denoted by red and blue colours, respectively. (**D**,**E**) Segregated activities of SN A and SN B in PB-responsive (D) and PLD-E-responsive *sw-*A2 sensilla (E). (**F**) Olfactory responses of SN A and SN B to the tested odourants. We selected 12 pheromone-responsive *sw*-A2 sensilla from our SSRs (sample numbers in F and Fig. 2D) and sorted spikes of SN A and SN B. M: male, F: female.

Among pheromone-responsive *sw*-A2 sensilla, three sensilla exhibited excitatory responses to both sex and aggregation pheromones (#4, #11 and #12 indicated in Fig. 2D). This result raises a question of whether a specific OSN that can process both sex and aggregation pheromones exists in *sw-*A2 sensilla. Therefore, we sorted spikes using specific electrophysiological data of pheromone-responsive *sw-*A2 sensilla, and revealed the response properties of each of the two OSNs in these sensilla (Fig. 3).

### Response properties of PB- and PLD-E-responsive SNs in *sw*-A2 sensilla

Small- and large-amplitude spikes from different OSNs were identical in each SSR from single *sw-*A2 sensilla of fourth instar nymphs (Figs. 2 and 3). We sorted these spikes using spike sorting software based on the spike amplitudes and durations (Fig. 3A–E) and successfully sorted into the two types of spikes from 12 pheromone-responsive *sw-*A2 sensilla (Fig. 3F). We termed large-spike and small-spike OSNs as “SN A” and “SN B”, respectively. PB exclusively activated SN B in PB-responsive *sw-*A2 sensilla, whereas PLD-E exclusively activated SN A in PLD-E-responsive sensilla (Fig. 3D,E). In the *sw-*A2 sensilla which responded to both pheromones, PLD-E-responsive SN A and PB-responsive SN B were co-localised (#11 and #12 in Fig. 3F). Thus, PB and PLD-E were independently detected by different OSNs, and we termed these as PB-SNs and PLD-E-SNs. None of PB-SNs responded to other tested odours (Fig. 3F). In contrast to the PB-SNs, PLD-E-SNs showed diverse response spectra; some PLD-E-SNs exhibited excitatory responses not only to PLD-E but also to general odours (#7 and #15 SN A in Fig. 3F).

**Figure 4:**
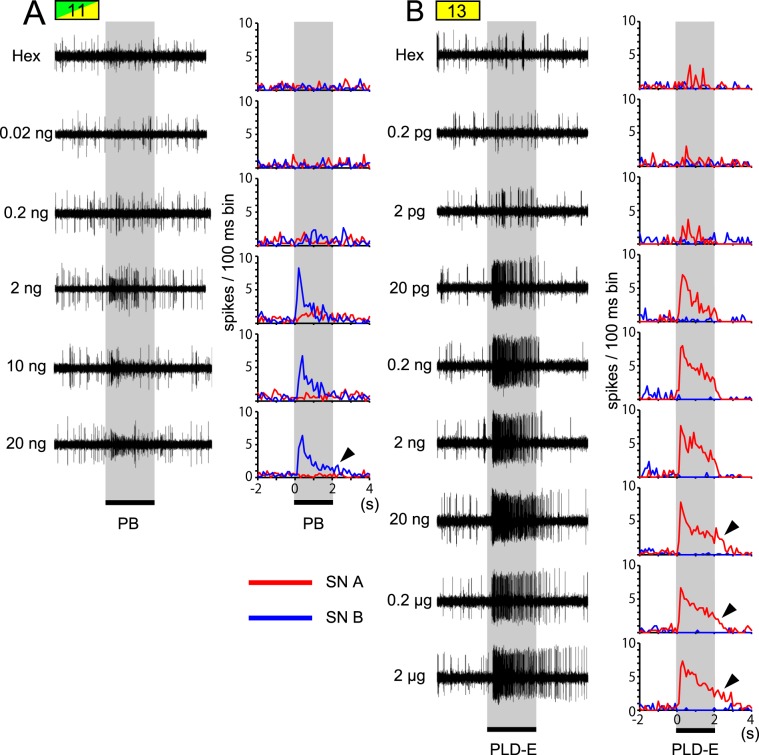
Temporal activity patterns of SN A and SN B that respond to sex and aggregation pheromones. (**A**,**B**) Temporal activity patterns of SN A and SN B to a given concentration of PB (**A**) and PLD-E (**B**). Olfactory responses (left panels) are obtained from a PB-responsive *sw*-A2 sensillum (A: #11 in Figs. 2D and 3F) and a PLD-E-responsive *sw*-A2 sensillum (B: #13 in Figs. 2D and 3F). Responses of SN A and SN B are displayed as peri-stimulus time histograms with 100 ms bins (right panels).

We observed that an OSN which paired with pheromone-responsive OSN in single *sw-*A2 sensilla was activated by general odours (Fig. 3F). In each *sw-*A2 sensillum, both SN A and SN B exhibited spontaneous spike activity and tended to be reciprocally inhibited by effective odours; the spike activity of an OSN was decreased during the period that the other OSN exhibited a high spike frequency (Figs. 3F and 4).

**Figure 5:**
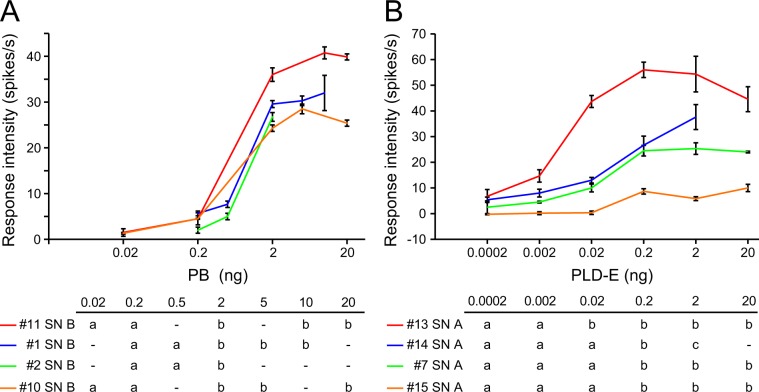
Dose-response curves of PB-SNs (**A**) and PLD-E-SNs (**B**). The averaged “R−R0” values of four PB-SNs (**A**) and four PLD-E-SNs (**B**) to a given concentration of pheromones are plotted with the standard error (vertical bars). Response spectra of four PB-SNs and four PLD-E-SNs are denoted in Fig. 3F (see sample numbers). In each of recordings, response intensities to different concentrations of pheromones are compared (one-way ANOVA and post-hoc Tukey’s test), and the same alphabet letters represent no statistical significant differences (P > 0.05).

Finally, we examined the dose-response relationships of PB-SN responses to PB and PLD-E-SN responses to PLD-E. In both PB-SNs and PLD-E-SNs, temporal response patterns to pheromones were changed from phasic to phasic-tonic according to the increase of concentrations (Fig. 4). The tonic phases elicited by high pheromone concentration lasted long beyond the stimulus periods (arrowhead in Fig. 4A,B). We evaluated the increase in spike frequency during the 1-sec period after stimulus onset (Fig. 5). All responses of PB-SNs typically showed sigmoidal dose-response curves and began to increase monotonically at 0.2 ng of PB and nearly reached a plateau at 2 ng (Fig. 5A). The curves indicated that the threshold concentration of PB lies between these concentrations (one-way ANOVA and post-hoc Tukey’s test, different alphabet letters; P < 0.05 in Fig. 5A). All recorded PB-SNs exhibited similar dose-response curves, but the dose-response curves to PLD-E varied across four PLD-E-SNs (Fig. 5B). The responses of the most sensitive PLD-E-SN began to increase when 2 pg of PLD-E was presented and reached a plateau at 20 pg (#13 SN A in Fig. 5B; one-way ANOVA and post-hoc Tukey’s test). Although the sensitivity of recorded PLD-E-SNs was various, as shown in Fig. 5B, threshold concentration of all tested PLD-E-SNs to PLD-E appeared to be higher than that of PB-SNs to PB. Interestingly, the maximal spike frequency widely varied from 15 to 55 Hz in PLD-E-SNs. In addition, the PLD-E-SN responded only to PLD-E (#13 SN A in Figs. 3F and 5B) exhibited higher sensitivity than those responded both PLD-E and general odours (#7 SN A in Figs. 3F and 5B). These results showed that there are several types of PLD-E-SNs that have different physiological properties in the nymphal antenna.

## Discussion

We electrophysiologically examined pheromone and odour detection in nymphal cockroaches and found that sex and aggregation pheromones are independently detected by distinct OSNs in the newly identified *sw-*A2 sensilla in nymphs. As far as we know, this is the first report on the cellular mechanisms of pheromone detection in nymphs of hemimetabolous insects. Sex and aggregation pheromone-responsive SNs exhibited high specificities and high sensitivities to their respective pheromones. Because the nymphal cockroaches lack *sw-*B sensilla, which have been confirmed to respond to sex pheromones in adults, and exhibits no behavioural responses to sex pheromones, it is surprising that nymphs possess high sensitive sex pheromone-responsive SNs. The behaviours elicited by sex and aggregation pheromones differ depending on the postembryonic developmental stages of the cockroach. Thus, the postembryonic developments of neural olfactory circuits and of their physiological properties are important factors for understanding expression of pheromone behaviours in hemimetabolous insects.

In this study, we classified nymphal and adult *sw-*A sensilla into *sw-*A1 and *sw-*A2 sensilla based on the external appearance of olfactory pores (Fig. 1). Different sizes and spatial patterns of olfactory pores in a given morphological type of olfactory sensilla have not been reported in other insects. In fruit flies, the formation of olfactory pores is controlled by a specific gene for cuticle secretion in *single-walled* olfactory sensilla^39^. Thus, olfactory pores of the cockroach might also be formed by specific gene control. Furthermore, *sw-*A1 and *sw-*A2 sensilla exhibited different olfactory response spectra, suggesting that OSNs in the two sensilla types might express different repertories of olfactory receptors. Therefore, *sw-*A1 and *sw-*A2 sensilla appear to have separate origins similar to the *single-walled* and *double-walled* olfactory sensilla, which exhibit different pore structures^40^.

**Figure 6:**
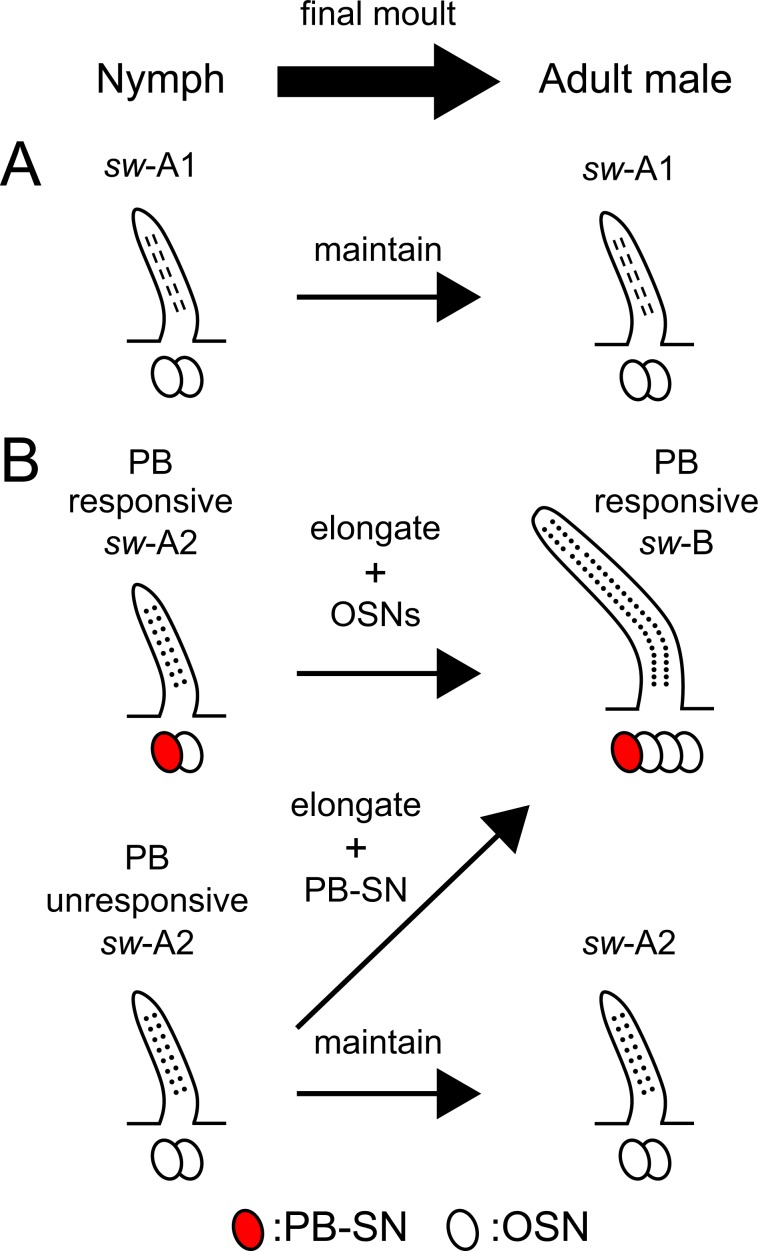
Leading models of the postembryonic developments of olfactory sensilla. (**A**) Schematic drawing of the postembryonic development of *sw*-A1 sensilla. Anatomical and physiological features indicate that nymphal *sw*-A1 sensilla are maintained during the postembryonic development of the cockroach. (**B**) Schematic drawing of the postembryonic development of sex pheromone-responsive sensilla. In this study, we obtained following results. (1) Both nymphal *sw*-A2 and adult *sw*-B sensilla have common shapes of olfactory pores. (2) All recorded nymphal *sw*-A2 sensilla have two OSNs, and there are PB-responsive and PB-unresponsive *sw*-A2 sensilla in nymphs. It has been known that each *sw*-B sensillum has one PB-SN and three other OSNs^17,19^. Furthermore, a large majority of nymphal *sw*-A sensilla transform to the *sw*-B sensilla at the final moult, and the morphogenesis is accompanied the increase of the number of OSNs^28^. Taken together current and previous results, we hypothesized developmental schemes of the sex pheromone-responsive sensilla.

Taken together with previous and current results, we hypothesized postembryonic developments of *sw*-A and *sw*-B sensilla in the cockroach (Fig. 6). In the male cockroach, at the final moult, a large majority of the nymphal *sw-*A sensilla transform into *sw-*B sensilla, and the remaining *sw-*A sensilla retain their morphology^17,28^. In this study, we revealed that adult *sw*-A sensilla also classified into *sw*-A1 and *sw*-A2 sensilla based on differences of olfactory pore shapes. The nymphal and adult *sw*-A1 sensilla share common morphological features, such as sensillar sizes, olfactory pores (Fig. 1C,D), number of OSNs^17^ and distribution patterns (Fig. 1A). In addition, OSNs in nymphal *sw-*A1 sensilla and a subset of adult *sw-*A sensilla have similar response spectra to general odours (Fig. 2D)^20,22^. It suggests that *sw*-A1 sensilla are morphologically and physiologically maintained during postembryonic developments (Fig. 6A). Interestingly, both adult *sw*-B and *sw*-A2 sensilla exhibited circular olfactory pores which identified in nymphal *sw*-A2 sensilla. SSR experiments revealed that nymphal *sw*-A2 sensilla had two OSNs and classified into PB-responsive and PB-unresponsive types. Whereas, in adult males, *sw-*B sensilla are always PB-responsive^19^ and each *sw-*B sensillum contains four OSNs including a PB-SN^15,17,22^. Because the morphogenesis from *sw*-A to *sw*-B sensilla is often accompanied by an increasing number of OSNs^28^, nymphal *sw-*A2 sensilla, especially PB-responsive *sw-*A2 sensilla, must transform into the adult *sw-*B sensilla (Fig. 6B). Therefore, PB-SNs in the nymphal *sw-*A2 sensilla might be inherited by the adult *sw*-B sensilla. Adult *sw*-A2 sensilla must be originated from PB-unresponsive *sw*-A2 sensilla (Fig. 6B). But, we can not deny the possibility that PB-unresponsive *sw*-A2 sensilla transform into the *sw*-B sensilla by acquiring the PB-SNs during the postembryonic development (Fig. 6B). It needs more detailed observations of sensillar morphogenesis during the postembryonic development.

The length of *sw-*B sensillum in the adult is about two-fold longer than that of a nymphal *sw-*A2 sensillum^17,28^. In accordance with the sensillar morphogenesis at the final moult, dendrites of PB-SNs expand^17^, which might result in a greater number of PB receptors on the dendrite and greater number of olfactory pores on cuticular apparatus. Therefore, PB-SNs in *sw-*B sensilla are hypothesised to exhibit higher sensitivity to sex pheromones than those in nymphal *sw-*A2 sensilla^17,28^. The response thresholds of PB-SNs in nymphal *sw-*A2 sensilla range between 0.2 and 2 ng of PB, and those in adult *sw-*B sensilla have been reported to range between 0.1 ng and 1 ng of PB^19^. Thus, the PB sensitivity of PB-SNs is not significantly different between nymphs and adults. However, other physiological features of PB-SNs significantly differ between nymphs and adults; PB-SNs in *sw-*B sensilla frequently adapt to higher concentrations of PB^19^, but this does not occur in nymphal *sw-*A2 sensilla. Further electrophysiological experiments are needed to determine the functional effects of morphogenesis from *sw*-A2 to *sw*-B sensilla.

Although both nymphal and adult cockroaches sense sex pheromones, the expression of sexual behaviours induced by sex pheromones is restricted to adults^10,11^. Sexual behaviours may be driven by physiological changes in pheromone-responsive neurons during postembryonic development because the major pheromone processing pathway from peripheral to higher brain centres is established during early development in the insect^29,30,41^. At the peripheral level, numbers of olfactory sensilla and sensory afferents including PB-SNs in the adult male antenna are more than 10-fold greater than those in the fourth instar antenna^17,27^. Reflecting the number of PB-SNs, the size of B-glomerulus in adult males is also approximately 250-fold greater than that of fourth instars^30^. Therefore, projection neurons innervating the B-glomerulus (B-PNs) in adult males may exhibit higher sensitivity to PB than that in fourth instar nymphs. In fact, in adult females which have a small number of *sw-*B sensilla and a small sized B-glomerulus that is equivalent in size to that of mid-instar nymphs^42^, B-PNs exhibited the weaker sensitivity to PB compared with those in adult males^43^. It suggests that female and nymphal B-PNs require higher concentrations of PB to generate responses equivalent to those of males. The functional significance of sex pheromones in nymphal cockroaches is still unknown, but nymphal cockroaches sufficiently detect and process higher concentrations of PB.

In this study, we identified olfactory sensilla and OSNs in nymphal cockroaches that detect the aggregation pheromone PLD-E. Aggregation pheromones have been behaviourally identified in many insect species^1^, but the neural processing mechanisms of the pheromone are still unknown. On the basis of the dose-response curves and response spectra of PLD-E-SNs, we can speculate that there are several types of PLD-E-SNs which express different olfactory receptors. Among them, PLD-E-SNs that are selectively tuned to PLD-E exhibited high sensitivity to this pheromone. Their low threshold for PLD-E is consistent with the behavioural threshold as shown in the olfactometer assay using the early instar cockroaches^14^.

There are 205 glomeruli in the antennal lobe of *P. americana* regardless of sexes and instar stages, and these loci are maintained throughout all instar stages, although their sizes are larger in the later instar nymphs^23,30,42^. As PB is processed by B-glomerulus in the later instar nymphs, adult males and females^24,34,43^, PB would be also processed in the B-glomerulus in the earlier instar nymphs. In contrast, glomerulus processing PLD-E in both adults and nymphs has not been identified. In adult AL, 205 glomeruli are clearly organized into the anterodorsal and the posteroventral groups on the basis of differences in their locations, morphologies, functions and developments^23,38,41,44^. Except for PB-SNs and PA-SNs, OSNs in *sw-*A and *sw-*B sensilla exclusivelly terminate in ordinary glomeruli in the anterodorsal group in adults^44^. If this feature is present in nymphs, PLD-E-SNs in *sw*-A2 sensilla might also project to given glomeruli of the anterodorsal group. Interestingly, in first to fourth instar nymphs of the cockroach, the nymphal B-glomerulus is smaller than other ordinary glomeruli in the anterodorsal group^30^. As glomeruli receiving many OSNs generally tend to be large in size^28,30^, the earlier instar nymphs may have a larger number of PLD-E-SNs than PB-SNs. In addition, PLD-E-SNs have a 100 times higher sensitivity than PB-SNs to their effective pheromones in fourth instar nymphs. Taking these results together, the nymphal olfactory system appears to be tuned to detection of the aggregation pheromone instead of the sex pheromone. This finding is correlated with the behaviour of nymphal cockroaches, especially early instars, in that they are strongly attracted to aggregation pheromones but not to sex pheromones^14^.

Both PB- and PLD-E-SNs tended to inhibit during the activated period of the other OSN in a given sensillum (Figs. 3F and 4). This inhibition among co-localised OSNs affects the detection of pheromones and general odours. In the Japanese beetle *Popillia japonica*, two OSNs in a sensillum, which detect a conspecific pheromone and heterospecific kairomone, inhibit each other and increase the perceived contrast between these two odours^45^. In fruits flies and mosquitoes, inhibition of co-localised OSNs in single olfactory sensillum is mediated by non-synaptic interaction and improves detection of the given odour stimulus^46,47^. Because both sex and aggregation pheromones are contained in the faeces, the cockroach receives these pheromones and many other odourants at the same time in the natural condition. Therefore, the strong activity elicited by pheromones might inhibit the activity of co-localized OSNs. Cockroaches tune peripheral system that can detect behaviourally salient pheromones from complex and cluttered odour environments.

## Materials and Methods

### Insects

Fourth instar males and females, and adult males of *P. americana* with intact antennae were used in this study. *P. americana* develops to the adult stage via 11 moults^27,28,30^. The cockroaches were obtained from laboratory colonies maintained at 28 °C under a 12:12 light-dark cycle at Fukuoka University. Since the aggregation pheromone PLD-E effectively attracts earlier instar cockroaches^14^, fourth instar nymphs were used for the electrophysiological study because of the technical limitations of SSR. The nymphal stages were unambiguously identified based on the lengths of the whole body, hind tibia and abdomen^48^. Sexes were discriminated by examining the morphologies of the last two abdominal sternites^49^. The criteria were applicable nymphs of *Periplaneta* species including *P. americana*^30,42,49^.

### Field emission scanning electron microscopy

After anesthetising nymphal and adult cockroaches on ice, whole antennae were isolated using a razor blade. The isolated antennae were immersed in 50% acetone and ultrasonically cleaned for 1 min. Antennae were dehydrated in an ascending acetone series (50% to 100%) and dried at 60 °C for more than 3 hours. Thereafter, each antenna was attached to an aluminium stub using water-soluble glue. After drying again, antennae were coated with platinum-palladium using an ion sputter (E-1045; Hitachi, Tokyo, Japan). Observations were performed using a field emission SEM (S-4800; Hitachi, Tokyo, Japan). After counting the number of flagellomeres, the external structures of the sensilla on a given flagellomere were thoroughly examined, and their digital images were obtained. The obtained images were processed using Adobe Photoshop CS3 and Illustrator CS3 (Adobe Systems, San Jose, CA, USA). Regarding the sensillar nomenclature, we referred to previous studies of adult *P. americana*^15,17,18,28^.

### Single sensillum recording

The method used for extracellular recording from OSNs in single sensilla of the nymphs was modified from the method used for adult *P. americana*^22^. In this study, the fourth instar males and females were used for the electrophysiological experiments. For a SSR, the ice-anesthetised nymph was immobilised ventral-side-up on an acrylic plate that had been covered with a thin layer of low-melting point wax. To prevent movement, the body and legs were gently mounted on the plate using low-melting wax, the neck was immobilised with small acrylic plates, and the antennae were gently fixed using the wax on the plate.

The plate was placed on the stage of a light microscope (AZ100, Nikon, Tokyo, Japan). The antenna was observed through the microscope at 500 × magnification. We performed SSRs from arbitrarily selected olfactory sensilla on the ventral surface of the flagellum. For recording, a silver wire indifferent electrode was manually inserted into the head capsule near the ipsilateral compound eye, and a tungsten recording electrode, which had been electrolytically sharpened in saturated KNO_2_ solution, was inserted into the basal cavity of the sensillum using a micromanipulator (Fig. 2A1–C1). After observing the spontaneous spike activities of OSNs, olfactory stimuli were presented to the antenna. Electrical signals were processed by a preamplifier with high input impedance (MEZ-8201; Nihon Kohden, Tokyo, Japan), amplified by a main AC/DC amplifier (EX-1; Dagan Corporation, Minneapolis, MN, USA), and displayed on an oscilloscope. Signals were digitised and recorded with a Power Lab data acquisition system at a sampling rate of 20 kHz (Power Lab 8/35; AD Instruments Japan Inc., Nagoya, Japan).

To identify the sensillum type, the recorded sensillum was observed with a SEM (Fig. 2A2–C2). After recording of olfactory responses, the recorded sensillum was marked by removing the surrounding bristles by manipulating the electrode. Then, an antenna fragment that contained the recorded sensillum was observed in detail using a SEM. The sensillar types were unambiguously identified based on the shape of the cuticular apparatus and olfactory pores as described in the result section.

### Olfactory stimulation

We used 15 different odourants and their solvents as well as purified substances of the main components of the sex pheromone (PB) and the aggregation pheromone (PLD-E) that were synthesised previously as odour stimuli (Table 1)^14,50^. Six odourants effectively attract nymphal cockroaches^5,35–37^, and eight odourants are used for classification of OSN types in adults^22^. Both PB and PLD-E were diluted in hexane at a concentration of 0.1 ng/µl as stock solutions. Faecal substances were extracted by immersing 5 g of dry faeces from cockroaches fed an agar/sugar diet in 10 ml of dichloromethane for 30 min (faecal extract)^13^. A gas chromatograph assay revealed that 16.6 µl of the faecal extract contained 2 ng of PLD-E (data not shown). Cuticular hydrocarbons were crudely extracted by immersing 40 fourth instar nymphs in dichloromethane for 1 hour (Cr-CHCs). Each odourant was dissolved in hexane or dichloromethane, placed on an aluminium plate (15 × 5 mm), and dried 3 min to evaporate the solvent. Therefore, their concentrations were denoted as dry weights in this study. Other odourants were diluted in paraffin oil at a concentration of 10 mM, and 20 µl of odourant solution was added to a piece of filter paper (15 × 5 mm). Immediately before recordings, the aluminium plates and the filter papers loaded with odourants were separately inserted into glass pipettes.

The odour stimulation device used in this study has been reported in a previous study^38^. Fresh air from outdoors was transported via a diaphragm air pump and was cleaned and dried with charcoal and silica-gel filters. The air stream was maintained at 1 l/min using a flowmeter. The main tube was connected to a three-way solenoid valve, which was operated by a stimulator (SEN7203, Nihon Kohden, Tokyo, Japan). During the inter-stimulus period, the constant air in the tube, which was connected to one outlet of the valve, passed through the blank glass pipette and flowed over the antenna. During stimulation period, the constant air stream was stopped and the air stream from the other outlet of the valve was passed through the glass pipette containing a given odourant. The tips of glass pipettes were positioned approximately 3 cm apart from the targeted sensillum. The air around the preparation was continuously exhausted through a duct behind the recording electrode. We regarded the timing of the solenoid valve gating as the onset of odour stimulation. The stimulus period was set to 1 or 2 sec. Each odourant was presented two to five times with >30 sec intervals. After stimulation, a new glass pipette containing another odorant was attached.

### Data analysis

In each SSR, several different shapes of spikes from different OSNs were concurrently recorded. Although the spike amplitudes were distinguishable in several traces, the spikes were not easily distinguishable in many recordings. Therefore, we grouped spikes with different shapes in each SSR and calculated the increase in total spike frequency from the spontaneous frequency as follows; R−R0, where R and R0 were the total numbers of spikes during the 1-sec period before and after the onset of odour stimulation, respectively. The average response was defined as the response intensity to a given odorant.

To evaluate the response properties of each OSN in single sensilla, we sorted spikes with different shapes in several recordings using the spike sorting function of Spike 2 ver. 8.08 (CED, Cambridge, UK). We performed principal component analysis on the recorded spikes according to the spike amplitude and duration. Each spike was plotted in a three-dimensional space using the first three principal components, and the spikes were clustered (Fig. 3C). The response intensity was represented as R − R0. Statistical analyses were performed using the free software R v.3.3.2 (R Foundation for Statistical Computing, Vienna, Austria).

## Data Availability

The electrophysiological datasets used in the current study are available from the corresponding author on reasonable request.
